# Insulin-like growth factor 1 receptor activation promotes mammary gland tumor development by increasing glycolysis and promoting biomass production

**DOI:** 10.1186/s13058-017-0802-0

**Published:** 2017-02-07

**Authors:** Bas ter Braak, Christine L. Siezen, Joo S. Lee, Pooja Rao, Charlotte Voorhoeve, Eytan Ruppin, Jan Willem van der Laan, Bob van de Water

**Affiliations:** 1 Division of Toxicology, Leiden Academic Centre for Drug Research, Leiden University, Einsteinweg 55, 2333 CC Leiden, The Netherlands; 2 Medicines Evaluation Board (MEB), Graadt van Roggenweg 500, 3531 AH Utrecht, The Netherlands; 3 The Blavatnik School of Computer Science, Tel-Aviv University, Tel Aviv, 69978 Israel; 4 ServiceXS, Plesmanlaan 1 /D, 2333 BZ Leiden, The Netherlands; 5 Centre for Health Protection, National Institute for Public Health and the Environment (RIVM), Antonie van Leeuwenhoeklaan 9, 3721 MA Bilthoven, The Netherlands

**Keywords:** Next-generation sequencing, Mammary gland tumor, IGF1R, Hallmarks of cancer, Warburg

## Abstract

**Background:**

The insulin-like growth factor 1 (IGF1) signaling axis plays a major role in tumorigenesis. In a previous experiment, we chronically treated mice with several agonists of the IGF1 receptor (IGF1R). We found that chronic treatment with insulin analogues with high affinity towards the IGF1R (IGF1 and X10) decreased the mammary gland tumor latency time in a p53^R270H/+^WAPCre mouse model. Frequent injections with insulin analogues that only mildly activated the IGF1R in vivo (glargine and insulin) did not significantly decrease the tumor latency time in this mouse model.

**Methods:**

Here, we performed next-generation RNA sequencing (40 million, 100 bp reads) on 50 mammary gland tumors to unravel the underlying mechanisms of IGF1R-promoted tumorigenesis. Mutational profiling of the individual tumors was performed to screen for treatment-specific mutations. The transcriptomic data were used to construct a support vector machine (SVM) classifier so that the phenotypic characteristics of tumors exposed to the different insulin analogue treatments could be predicted. For translational purposes, we ran the same classifiers on transcriptomic (micro-array) data of insulin analogue-exposed human breast cancer cell lines. Genome-scale metabolic modeling was performed with iMAT.

**Results:**

We found that chronic X10 and IGF1 treatment resulted in tumors with an increased and sustained proliferative and invasive transcriptomic profile. Furthermore, a Warburg-like effect with increased glycolysis was observed in tumors of the X10/IGF1 groups and, to a lesser extent, also in glargine-induced tumors. A metabolic flux analysis revealed that this enhanced glycolysis programming in X10/IGF1 tumors was associated with increased biomass production programs. Although none of the treatments induced genetic instability or enhanced mutagenesis, mutations in *Ezh2* and *Hras* were enriched in X10/IGF1 treatment tumors.

**Conclusions:**

Overall, these data suggest that the decreased mammary gland tumor latency time caused by chronic IGF1R activation is related to modulation of tumor progression rather than increased tumor initiation.

**Electronic supplementary material:**

The online version of this article (doi:10.1186/s13058-017-0802-0) contains supplementary material, which is available to authorized users.

## Background

The insulin-like growth factor 1 (IGF1) pathway plays a critical role in cell growth, cell survival, and protection from apoptosis. It is therefore not surprising that epigenetic and transcriptional changes in IGF1 signaling can induce cancer development and progression [1, 2].

The administration of exogenous insulin is a common therapy for types I and II diabetes. New insulin-like molecules have small modifications of the insulin molecular structure to improve pharmacokinetic parameters of the original molecule and thereby increase stability and temporal bioavailability. These molecular changes possibly affect the binding affinity towards receptors including the insulin receptor (IR) or the IGF1 receptor (IGF1R). Consequently, insulin analogues with an increased IGF1R affinity likely promote mitogenesis [3]. Insulin X10 is an insulin analogue which possesses a well-known increased affinity towards the IGF1R and which consequently could promote tumorigenesis (for this reason insulin X10 has never entered the pharmaceutical market). Insulin glargine, currently the most commonly prescribed insulin therapy worldwide [4], also has an increased binding affinity towards the IGF1R. In vivo glargine is rapidly degraded into compounds with a lower affinity towards the IGF1R. Even today there are some concerns about the carcinogenic risk that the use of glargine might induce [5, 6].

To study the role of IGF1R signaling in breast cancer, we recently evaluated the tumor promoting capacity of chronic insulin analogue treatment in a human relevant breast cancer (p53^R270H/+^WAPCre) mouse model [7]. In this model, the WAPCre system ensures mammary gland (MG)-specific expression of the heterozygous p53 mutation, which corresponds to a mutational hotspot often found in patients with the *Li Fraumeni* cancer syndrome [8]. Eventually, all mice spontaneously developed these human relevant MG tumors within approximately 1 year. Chronic treatment with compounds that possess a high affinity towards the IGF1R, IGF1 and the insulin analogue X10, significantly decreased the tumor latency time. Frequent injections with insulin glargine, a compound that only mildly activates the IGF1R in vivo [5], showed a similar trend but the observed tumor latency time decrease was not significant compared to regular insulin [7]. Systematic signaling pathway mapping of all tumors revealed that the MAPK/ERK signaling cascade was especially strongly activated in IGF1- and X10-induced tumors. Although this provides some insight in to the alternative signaling wiring in insulin analogue-related tumors, a systematic evaluation of the genetic modifications and consequently alterations in cellular pathways and network biological differences of insulin analogue-related tumors is still obscure.

In this study, to gain more insight in to the modulation of tumor development and progression by chronic IGF1R activation, we used a systematic in-depth next-generation sequencing (NGS) approach. RNAseq analysis was performed on 50 insulin analogue-induced MG tumors (control, insulin, IGF1, X10, and glargine treatment). Overall genetic modifications were determined at a tumor level. NGS transcriptome analysis did shed light on the specific tumor development and progression in relation to chronic IGF1R activation. For this, we specifically evaluated the alternative modulation of the hallmarks of cancer [9] to detect treatment-specific tumor features.

## Methods

### Chronic in vivo insulin analogue treatment

Previously, we have reported the effect of insulin analogues on tumor development [7]. Here, for the chronic exposure experiment, 200 (40 mice per treatment), 8-week-old female p53^R270H/+^WAPCre mice were obtained from an in-house breeding project. The point mutation in the tumor suppressor p53 gene corresponds to the *Li Fraumeni* cancer syndrome mutational hotspot (R273H) in humans. Every other day these mice have been injected (subcutaneously) with either vehicle, insulin, glargine, X10, or IGF1 until tumor development. Once the tumors reached a size of 1 cm^3^ and 24 h after their last injection the mice were sacrificed. Tumors and other tissues were isolated. For this study, ¼ of the tumor was stored in RNALater (Ambion, Austin, Texas) at 4 °C for RNA isolation. A miRNA isolation kit (Macherey Nagel, Germany) was used to isolate and purify small and large RNA molecules in one fraction. Tumor latency time is defined as the time (in weeks) for the tumor to form, from the start of the experiment to the first time the tumor was palpated.

### Single insulin analogue treatment: an animal experiment

To determine the short-term effects of insulin analogue treatment on mammary gland gene expression, a single insulin analogue exposure experiment was performed with 40 (4 mice per treatment/time point) female, 8-week-old inbred FVB/NRj mice (obtained from Janvier, rodent research models, France). This specific mouse strain was used as it is the closest relation to the p53^R270H/+^WAPCre mouse strain. Mice received a single subcutaneous injection with either vehicle, insulin, glargine, X10, or IGF1. The mice were sacrificed 1 or 6 h after the injection, blood was collected (mini collect, Greiner/Omnilabo), and MGs were stored in RNALater (Ambion, USA) at 4 °C for RNA isolation. For this, a Nucleospin RNA isolation kit (Macherey Nagel, Germany) was used. For further technical details, please refer to our previous publication [7].

### In vitro stimulation experiments

Next, an in vitro experiment was performed to reveal the transcriptomic effects of insulin analogue exposure on a human breast cancer cell line. MCF7 human breast cancer cells with an overexpression of the IGF1R and a stable knockdown of the IR (MCF7 IGF1R) were seeded at 60% confluence and starved for 2 days in 5% charcoal/dextran-treated FBS (CDFBS, Hyclone, USA) containing RPMI 1640 (Gibco, USA) medium. Cells were stimulated (with 10 nM compound) for 1 or 6 h after which RNA was isolated using NucleoSpin® miRNA isolation kit (Machery Nagel, Düren, Germany). Stimulations included: insulin NPH (Insuman Basal, Sanofi Aventis), insulin glargine (Lantus, Sanofi Aventis), insulin X10 (AspB10, Novo Nordisk), and IGF1 (Increlex, Ipsen). For more technical details about cell line generation and characterization or growth factor stimulation, please refer to our previous publication [7].

### Next-generation sequencing and gene expression analysis

For the chronic insulin analogue exposure experiment, the quality and integrity of the RNA samples were analyzed using the bioanalyzer with an RNA nanochip. The Ion Total RNA-Seq kit was used to process the samples. Samples were Poly-A selected prior to library preparation. This library preparation included the cDNA synthesis and purification steps with the Ion Total RNA-Seq kit v2 (Life Technologies, UK) according to the manufacturer’s instructions. The Ion PI Template OT2 200 Kit v3 and Ion Sequencing 200 kit v3 (both Life Technologies) were used according to the manufacturer’s instructions for sequencing libraries on the PI chip. Sequence runs were performed on the Ion Proton Sequencer (at ServiceXS, Leiden). PI chip analysis, base calling, and quality checks were performed using the Torrent Server Suite. On average, 40 million reads per sample were sequenced with an average read length of 100 base pairs. No additional trimming or filtering of reads was performed before processing. Reads were aligned to mouse genome build GRCm38-Ensembl using Tophat2 (Version 2.0.10). Reads which could not be aligned using Tophat2 were aligned in an additional step, using Bowtie2 (Version 2-2.10) in the local, very sensitive mode. Tophat2 and Bowtie2 aligned reads were merged into a single .bam file for each sample before further analysis.

Gene expression was quantified using HTSeq-Count (Version 0.6.1) using the default options. Differential gene expression was analyzed for compound versus vehicle treatment and was performed using DESeq2 (Version1.2.10). For this analysis, genes with a read count of <50 reads across samples (average of <1 read per sample) were filtered out before the analysis. For the estimation of individual exon expression analysis, a RPKM table was generated with the read counts normalized to library size and gene length (using DEXSeq version 1.8.0). For the mutational profiling, the reads (unfiltered and untrimmed) were aligned to mouse genome build GRCm38.73-DNA primary assembly using TMAP within the Torrent Suite version 4.0.2. Variant calling was performed using the Torrent Suite Variant Caller version 4.0-r72612 with the settings tuned for the detection of somatic mutations at a low stringency level. The reference genome used was the same as that used for read alignment—GRCm38.73. SnpEff version 3.6c was used to filter and annotate the mutations. The list of mutations was filtered to include only exonic mutations with a quality score higher than 250. Several mutations are found in the exact same position in all tumor samples, probably strain-specific single nucleotide polymorphisms (SNPs). Known SNPs in coding regions for the mouse strain FVB/NJ (the strain most closely related to the ^p53R270H+/–^WAPCre) were downloaded from the Mouse Genome Informatics database and these mutations were discarded from the list. Mouse homologs of the list of human tumor driver genes [10] were used to define the most clinically relevant mutations.

### Phenotypic prediction based on transcriptomic data

To predict the phenotypic characteristics of the treatments of different insulin analogues using their transcriptomics, we constructed a support vector machine (SVM) classifier. We followed a procedure similar to that used in [11] to identify genes whose expression is significantly associated with cancer cell migration and proliferation across 52 breast cancer cell line data. We then applied them to the orthologs of the mouse transcriptomics data to predict the migratory and proliferative potential of 50 mouse mammary tumor samples using LIBSVM [11].

### Genome-scale metabolic modeling analysis

We estimated the metabolic fluxes that are most consistent with the transcriptomics data using a computational framework called iMAT [12]. iMAT integrates the transcriptomics, as ‘soft’ constraints, by ternary partitioning the expression to lowly (–1), mediocrely (0), and highly (1) expressed genes. iMAT then attempts to collect the metabolic states that best correspond to these cues, which constructs a mixed integer linear programming (MILP) problem. We applied iMAT to the human genome-scale metabolic model Recon1 [13] with a standard medium condition (RPMI) in a condition-specific manner for the five different insulin analogues treatments. The iMAT predicts (i) the biomass production rate and (ii) the metabolic flux rates of individual metabolic reactions. The biomass production rate is the rate at which the biomass precursors are generated with appropriate proportions, and it is incorporated in the metabolic network model as a putative reaction that takes cell and energetic requirements needed to produce biomass as input [14]. With the metabolic fluxes predicted by iMAT, we performed a pathway enrichment analysis of differentially activated metabolic reactions in X10/IGF1-treated cells to the remainder conditions (insulin/glargine/vehicle). We selected the metabolic pathways whose reactions are significantly enriched in the up-/downregulated group using a hypergeometric test followed by multiple hypotheses correction with the false discovery rate (FDR) 0.05. The predicted biomass production rate does not involve standard deviation because we focused on the metabolic states where the biomass production rate is optimized (thus single-valued). 

### Statistical analysis

Graphpad Prism version 5.01 software was used for the statistical analysis. All standard error bars in the graphs represent standard deviations. Unpaired two tailed *t* tests were performed to calculate significance. Multi-experiment viewer (MeV version 4.8.1) was used for the hierarchical clustering analysis.

## Results

### Genetic profiling of IGF1R signaling-mediated mammary gland tumor formation reveals cellular processes associated with decreased tumor latency

**Fig. 1:**
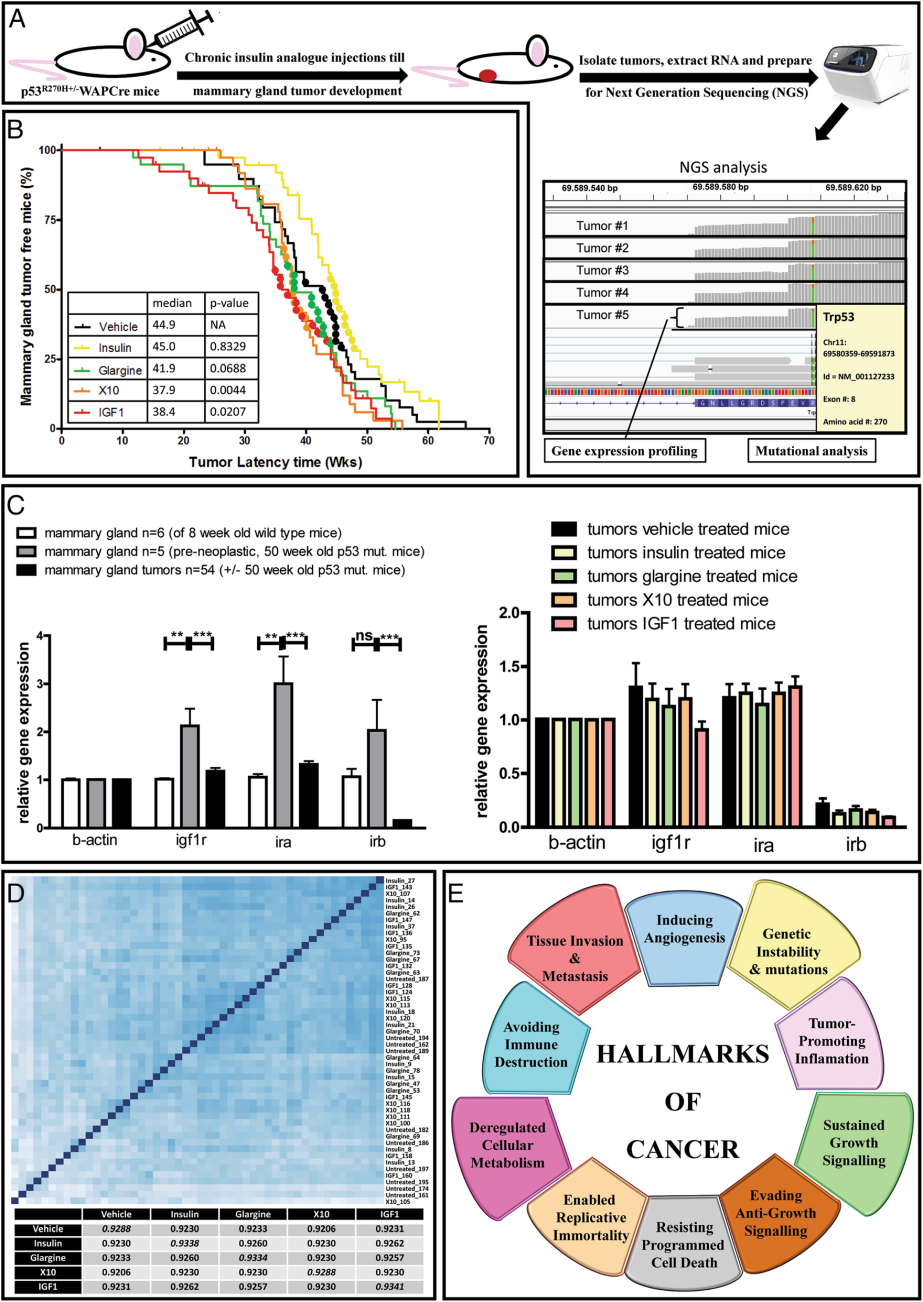
Experimental overview. **a** Overview of the chronic insulin analogue exposure experiment. **b** The Kaplan-Mayer MG tumor-free mice plots with the median tumor latency per treatment group; the colored dots indicate the selected tumors for the transcriptomic analysis. **c** the insulin-like growth factor-1 receptor (*igf1r*), A isoform of the insulin receptor (*ira*), and B isoform of the insulin receptor (*irb*) gene expression levels in MG tissue of young mice (8 weeks), old mice (~50 weeks), and in MG tumors (first graph); the second plot (*right*) shows the receptor gene expression distribution in the MG tumor tissue of mice chronically exposed to different insulin analogues. **d** Heat map of the next-generation sequencing data showing hierarchical sample clustering by sample-to-sample distance; the lower table shows the Spearman rank correlation coefficients within the treatment groups (*bold*) and the coefficient between the different treatment groups (averaged per condition). **e** The hallmarks of cancer with the features highlighted that we will discuss in view of the chronic insulin analogue exposure experiment. **P < 0.01, ***P < 0.001. *ns* not significant. (Adapted from Hanahan and Weinberg [9])

Previously, we chronically treated p53^R270H/+^WAPCre mice with insulin and insulin analogues to study the effect of chronic IGF1R stimulation on tumorigenesis (Fig. 1a). We found that MG tumor latency time in X10 and IGF1 (compounds with a high affinity towards the IGF1R) treatment groups was significantly decreased (Fig. 1b). Tumors in mice treated with glargine (an insulin analogue that induces only a mild IGF1R activation in the MG) also developed earlier compared to vehicle- and insulin-treated animals, but this trend was not significant [7]. To further investigate this, we evaluated receptor gene expression levels. We found that gene expression levels of the insulin receptor (both the A and B isoform; *ira*, *irb*, respectively) and insulin-like growth factor 1 receptor (*igf1r*) are significantly upregulated in pre-neoplastic MG tissue of old (50 weeks on average) p53^R270H/+^WAPCre mice compared to healthy 8-week-old MGs (Fig. 1c). Also, *ira* and *igf1r* levels were slightly upregulated in MG tumors. Interestingly, we found a significant and sevenfold downregulation of the B-isoform of the insulin receptor in MG tumor tissue compared to normal MG tissue. This effect was not treatment specific (Fig. 1c right). Levels of *igf1r* were only decreased after chronic treatment with IGF1. These data are indicative for an involvement of INSR/IGF1R signaling in tumor development and/or progression. However, the different receptor distributions cannot explain the differences in tumor latency time, since this was not a treatment-related effect.

To gain more insight in to overall differences between various insulin analogues in tumor development and progression, we performed RNAseq NGS of 50 tumors (10 tumors per treatment group). We reasoned that transcriptome analysis would shed light on the cause for the differences in tumor latency time by chronic insulin analogue treatment. From the 40 tumors per treatment group, 10 epithelial to mesenchymal transition (EMT)-like tumors were selected per treatment condition for the transcriptome analysis; these 10 EMT tumors had a tumor latency time that was closest to the median latency time of that specific entire treatment group. The selected tumors have been indicated with colored dots in the Kaplan-Mayer curve (Fig. 1b). In Fig. 1d, a correlation analysis shows the sample-to-sample distance. All transcriptomics data were used to define the correlation within and between the different treatment groups. In spite of the relatively high variation in this in vivo experiment, there was a higher correlation within each treatment group than between the different treatment groups. This suggests the existence of a treatment-specific response under each treatment condition. To define these treatment-specific responses, we focused on four particular hallmarks of cancer (Fig. 1e) that might have been involved in the decreased tumor latency, either by tumor initiation or progression. These pathways were selected in close relation to the signaling pathways downstream of IR and IGF1R, and included: i) sustained growth signaling; ii) tissue invasion and metastasis; iii) deregulated cellular metabolism; and iv) genetic instability and mutations.

### Chronic IGF1R activation of the MG results in tumors with a ‘sustained growth signaling’ signature

**Fig. 2:**
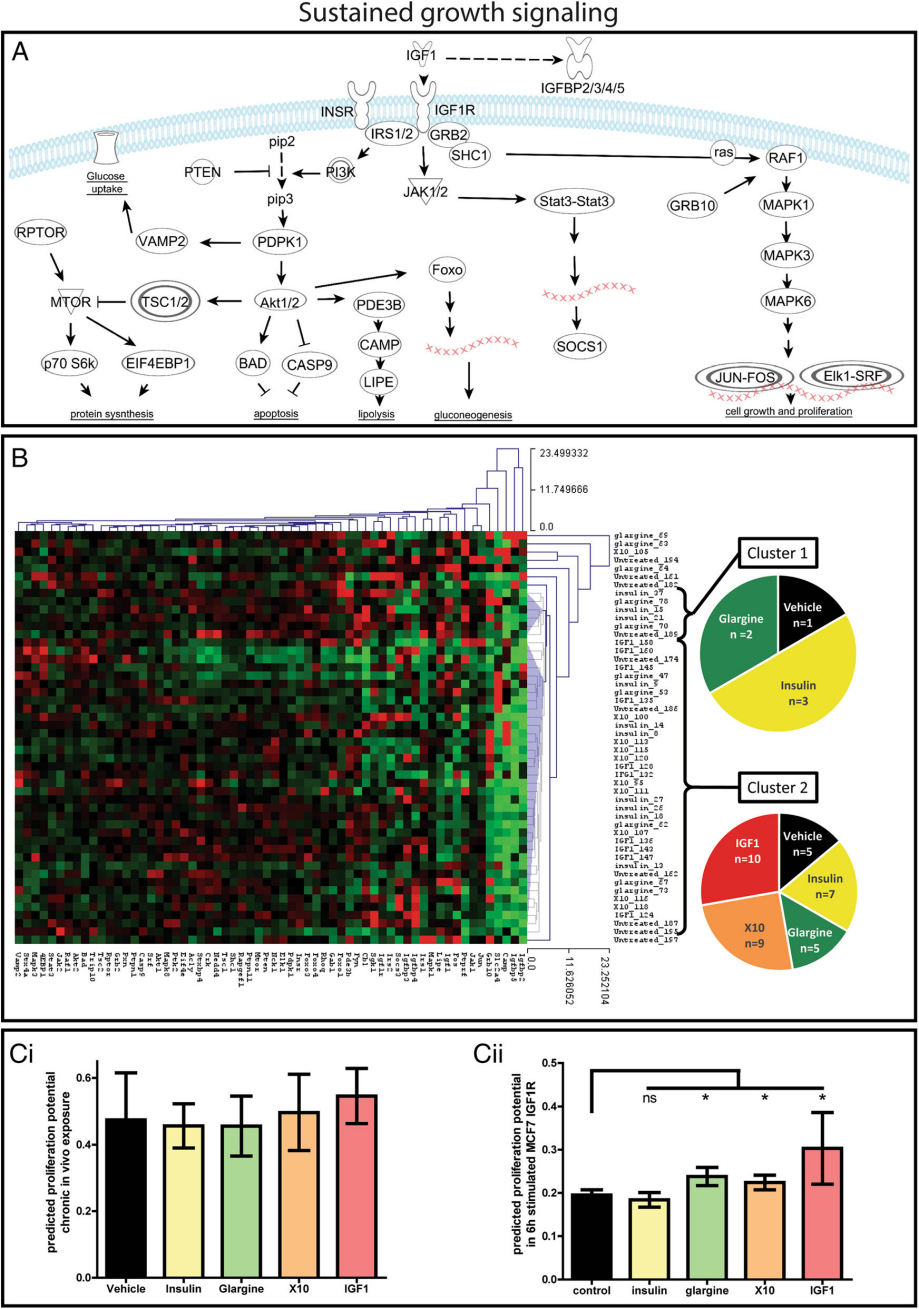
Sustained growth signaling in mammary gland tumor tissue of chronically insulin analogue exposed mice. **a** The INSR/IGF1R signaling pathway with receptors, downstream targets, and the biological effect. **b** Hierarchical clustering (Euclidian distance) of the INSR/IGF1R-specific gene expression per MG tumor. The pie diagrams show the distribution of the different treatments in the two clusters. **c** Bar graph of SVM simulation on the predicted proliferation potential per treatment of MG tumors of the chronically exposed mice (i) and human breast cancer cells (MCF7 IGF1R) exposed for 1 h to the indicated insulin analogue (ii). *P < 0.05. *ns* not significant

Proliferative signaling is normally a highly regulated process. In a tumor, the replicative cell homeostasis is deregulated causing sustained growth signaling [9]. Since mice were chronically exposed to insulin analogues that activate (to a lesser or greater extent) the IGF1R, we postulated that the decreased tumor latency time (after X10/IGF1 stimulation) would be a direct result of an upregulated IGF1R signaling pathway. We incorporated mouse orthologs of all human genes (~60 genes) that are directly involved in “INSR and IGF1R signaling” according to Ingenuity Pathway Analysis (IPA) (Fig. 2a). A hierarchical clustering analysis was performed for all 50 MG tumors on the relative expression levels of these genes (Fig. 2b). Two clusters could be defined. In cluster 1, tumors from low mitogenic treatments are enriched, whereas cluster 2 predominantly consists of the X10/IGF1 (high mitogenic compound) treated tumors. These two different clusters could not be linked to specific signaling pathways (e.g., PI3K, MAPK, JAK signaling cascade).

To predict the proliferative potential of all the individual tumors, we next built an SVM classifier using transcriptomic signatures associated with proliferation (Fig. 2ci). No significant effect was observed; however, a trend for the tumors in the IGF1 treatment groups to have an increased proliferative potential could be seen. As a positive control for the SVM simulation, similarly we ran the SVM simulation on our earlier micro-array data of the MCF7-IGF1R cells stimulated with the different insulin analogues. We observed that 1 h after glargine, X10, and IGF1 stimulation a strong and significant proliferative genetic signature could be detected. These results are in line with the proliferative potential as determined with functional in vitro assays using this cell line and insulin analogue exposures [5], which makes us believe that the transcriptomic profiles and used SVM simulation is predictive for the biological effect.

An effort was made to determine the growth rate of the individual tumors by calculating the tumor volume from time of detection until the time of sacrifice (data not shown). The EMT tumors in general were highly proliferative resulting in a very small timeframe (a couple of days to weeks) in which tumor volume could be assessed. Unfortunately, this resulted in just a few data points per tumor. Knowing that tumor growth is very likely not linear during tumor development we did not feel comfortable sharing these results. The proliferative potential was also assessed with ki67 staining on several tumors, but as expected there appeared to be a very small window and all tested tumors had a ki67 score >45% indicative of being highly proliferative (data not shown). Next, we attempted to isolate cells from each tumor; we anticipated that monitoring the growth rate of each mouse mammary gland tumor-derived cell line would resemble the growth rate of its originating tumor. Although it appeared to be rather easy to generate a cell line from each individual tumor, it was quite evident that just a small subset of cells would grow out from these tumors. We feared that this clonal expansion would give a skewed image of true tumor growth, and therefore we decided not to include these data.

### Chronic IGF1R activation induces tumors with a more mesenchymal phenotype and a higher migration potential

**Fig. 3:**
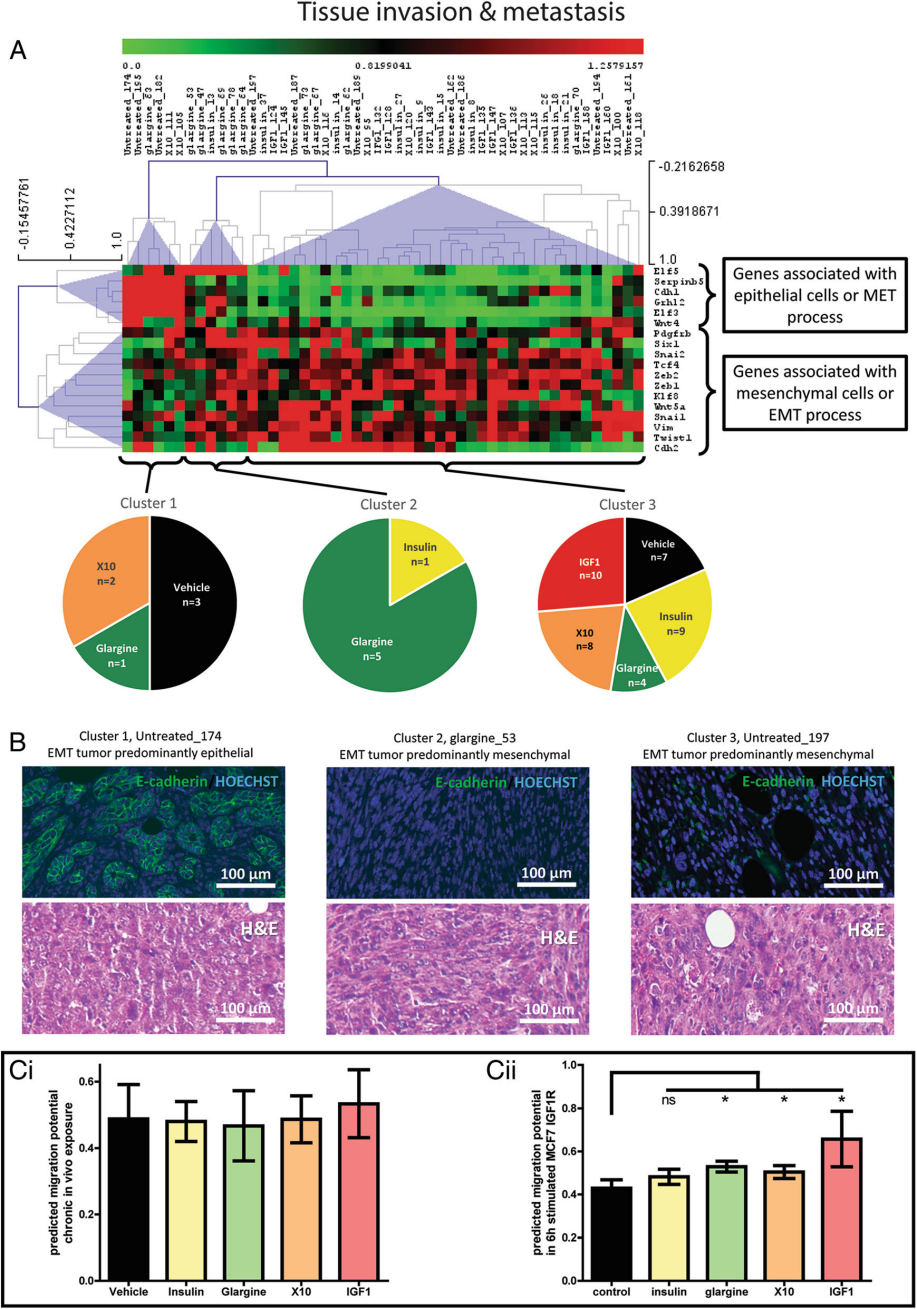
Tissue invasion and metastasis in mammary gland tumor tissue of chronically insulin analogue exposed mice. **a** Hierarchical clustering (Pearson correlation) of genes involved in epithelial to mesenchymal transition (EMT) or mesenchymal to epithelial transtition (MET) per MG tumor. The pie diagrams show the distribution of the different treatments in the three clusters. **b** The E-cadherin/HOECHST immunofluorescent hematoxylin and eosin (H&E) pathology slides of three example tumors showing epithelial or mesenchymal characteristics. **c** Bar graph of SVM simulation on the predicted migration potential per treatment of MG tumors of the chronically exposed mice (i) and human breast cancer cells (MCF7 IGF1R) exposed for 1 h to the indicated insulin analogue (ii). *P < 0.05. *ns* not significant

Next, we assessed the hallmark “tissue invasion and metastasis”. We calculated the average number of tumors per mouse per treatment group. Interestingly, we found a significantly higher number of tumors in the IGF1 group (and a non-significant increase for the chronic X10 and glargine treatment groups) compared to the vehicle-treated mice (Additional file 1). This effect can be both due to an increased incidence of multiple primary tumors as well as an increased formation of metastasis in the IGF1 group. To assess the underlying genetic pathways that might have induced metastasis formation we incorporated all genes that are directly associated with the EMT or the mesenchymal to epithelial transition (MET) according to IPA. The gene expression of the mouse orthologs of these genes is presented in an unsupervised hierarchical clustering (Fig. 3a). Two clear gene groups were observed: group A containing genes associated with epithelial phenotype (*Elf5*, *Serpinb5*, *Cdh1*, *Grhl2*, *Elf3*, and *Wnt4*), and group B containing twelve genes associated with mesenchymal cells (*Pdgfrb*, *Six1*, *Snai2*, *Tcf4*, *Zeb1*, *Klf8*, *Wnt5a*, *Snail*, *Vim*, *Twist1*, and *Cdh2*). From this hierarchical clustering, three separate treatment clusters could be discriminated. Cluster 1 consists of tumors that show high expression of epithelial- and low expression of mesenchymal-associated genes; three out of six tumors originate from the vehicle-treated animals. Cluster 2 shows moderate expression levels of epithelial- and mesenchymal-associated genes; interestingly the majority of these tumors were from the glargine treatment condition. Cluster 3 consists of tumors that have a low expression of epithelial and high expression of mesenchymal markers. IGF1 treatments were not present at all in cluster 1 and 2, while IGF1 and X10 treatment groups were over-represented in cluster 3. This suggests that IGF1R-mediated signaling is a driver of tumors with an EMT phenotype. We substantiated the phenotypes for the three different clusters (Fig. 3b). The tumors in cluster 1 predominantly expressed epithelial cells that contain many E-cadherin-positive cells. Tumors of the cluster 2 and 3 phenotype were characterized as EMT tumors predominantly consisting of mesenchymal cells, lack of E-cadherin staining, and clear smooth muscle actin (SMA) staining (data not shown) [7].

To link the transcriptomics data further to the phenotype we performed a SVM simulation to derive predictions for the cell migratory potential of each tumor (Fig. 3ci). Overall, no significant difference in the migration potential was observed for the different treatment groups; potentially the intrinsic high variation of in vivo experiments and smaller group sizes can explain lack of power in this analysis. To evaluate whether IGF1 signaling itself is a strong activator in vitro, we also applied our SVM classifier to our previous MCF7-IGF1R transcriptomics data exposed to the different insulin analogues (Fig. 3cii). IGF1, X10, and glargine caused a significant increase in the migration potential. Altogether, these data suggest that chronic exposure to X10, and especially IGF1, induces tumors with a more mesenchymal phenotype that are possibly more aggressive in terms of their migratory potential.

### Mice receiving a chronic X10 and IGF1 treatment develop tumors with a higher Warburg potential

**Fig. 4:**
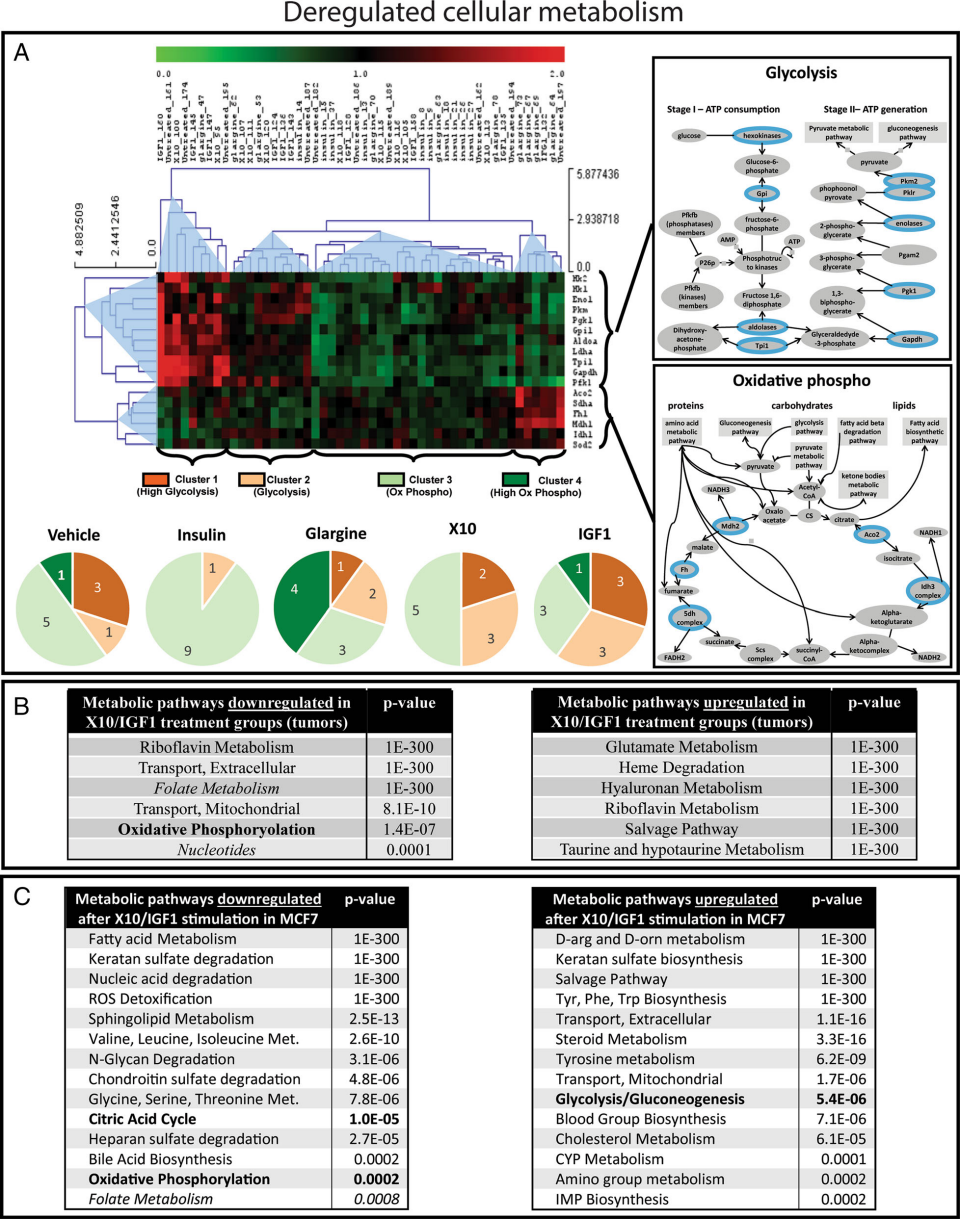
Warburg effect in mammary gland tumor tissue of chronically insulin analogue treated mice. **a** Hierarchical clustering (Pearson correlation) of genes involved in glycolysis or oxidative phosphorylation per MG tumor. The pie diagrams show the distribution of the different clusters per treatment group. **b** Table with the metabolic pathways that were significantly down- or upregulated in the X10/IGF1 treatment groups compared to the vehicle, insulin, and glargine treatment groups. **c** Table with the metabolic pathways that were significantly down- or upregulated after X10/IGF1 exposure compared to vehicle, insulin, and glargine treatment in the MCF7 IGF1R model

Through the activation of the INSR, insulin and insulin analogues directly affect cell metabolism. Therefore, we wondered whether the chronic treatment with insulin analogues induce tumors with a deregulated cellular metabolism or a higher Warburg potential. To test this we used all genes directly involved in “glycolysis” and in “oxidative phosphorylation” according to IPA. An unsupervised hierarchical clustering of the expression profiles of each tumor revealed two very clear gene groups (Fig. 4a). Strikingly, the first gene group consists of eleven genes that are all directly involved in glycolysis; all the genes in the second cluster are involved in oxidative phosphorylation. Four clear tumor clusters could be detected: cluster 1 with high expression of glycolysis genes and low expression of oxidative phosphorylation genes; cluster 2 with moderate glycolysis; cluster 3 with little glycolysis and moderate oxidative phosphorylation; and cluster 4 with high oxidative phosphorylation and little glycolysis. For the insulin group almost all tumors were related to cluster 3 (little glycolysis and moderate oxidative phosphorylation: 90%). For glargine-related tumors, 70% showed enrichment for genes regulating oxidative phosphorylation. IGF1 and X10 tumors showed increased expression of genes involved in glycolysis (60% and 50%, respectively) compared to the vehicle control. This suggests that chronic insulin signaling drives tumors in a more oxidative phosphorylation mode.

Since IGF1 and X10 were mostly related to glycolysis we performed an independent bioinformatics analysis on the activity of various metabolic processes. This revealed several significantly down- and upregulated metabolic programs in IGF1/X10-derived tumors compared to the vehicle/insulin/glargine tumors (Fig. 4b). Importantly, as expected, oxidative phosphorylation was significantly downregulated in the IGF1/X10 group (*P* = 0.000107) while glycolysis was significantly upregulated. Again, this analysis was also performed on the microarray data of MCF7-IGF1R cells exposed to the different insulin analogues. Similar to the in vivo data, IGF1 and X10 treatment significantly downregulated the citric acid cycle as well as oxidative phosphorylation (both *P* < 0.0005) and upregulated glycolysis (*P* < 0.00001) (Fig. 4c). These data suggest that both IGF1 and X10 promote a Warburg effect in mammary gland tumors (chronic exposure) and human tumor cell lines (a single 1 h exposure).

### Biomass production rate is highly upregulated in tumors of chronic X10/IGF1-treated mice

**Fig. 5:**
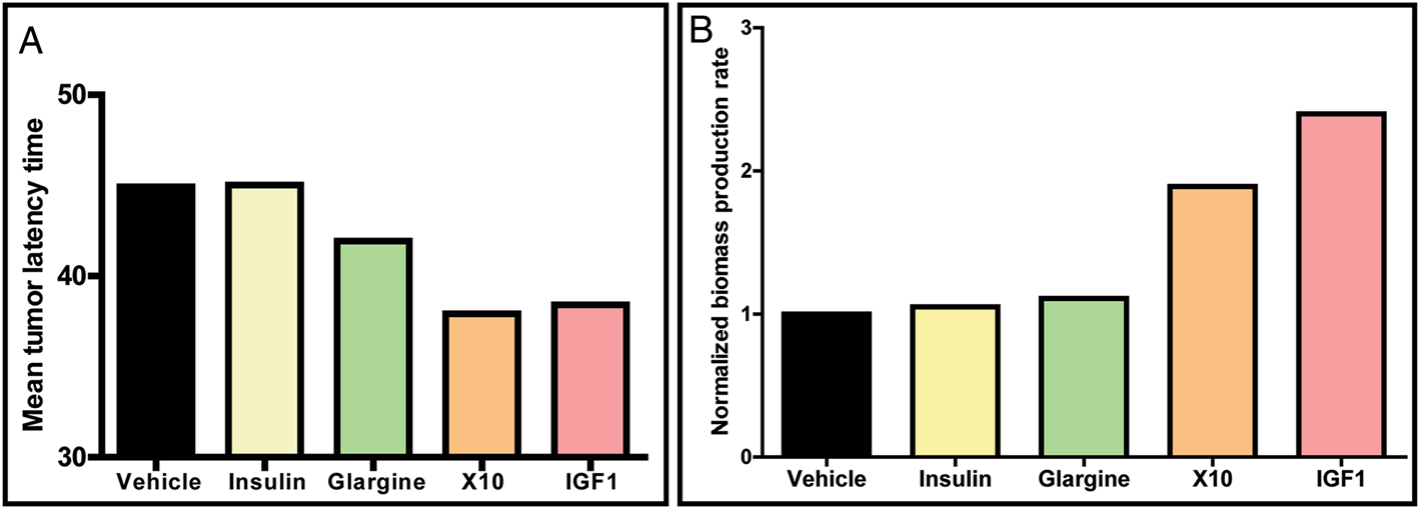
Predicted biomass production rate is increased in tumors of chronically X10/IGF1-treated mice and could possible explain the decreased tumor latency time in these treatments. **a** Bar plot of the mean tumor latency time in weeks per chronic treatment. **b** Bar plot of the normalized biomass production rate per treatment

Tumor mice receiving a chronic X10 and IGF1 treatment showed increased expression of glycolysis related genes (Fig. 4) and are associated with a decreased tumor latency time (Fig. 5a). We evaluated whether this difference in metabolic capacity is related to enhanced growth. To test this hypothesis, we predicted the rate of the accumulation of biomass in all these different tumors based on the gene expression profiles of biomass-producing metabolic processes. In Fig. 5b, a bar graph of these results is presented. A highly increased mammary gland tumor biomass production rate was observed in chronic X10- and IGF1-treated tumors. This finding is in agreement with the observed increased proliferative potential in the X10/IGF1 tumors. We hypothesize that the decreased tumor latency time by X10 and IGF1 treatment was caused by enhancing the tumor development rather than interfering with the tumor initiation.

### Ezh2 and Hras mutations are enriched in chronic IGF1R-activated tumors

**Fig. 6:**
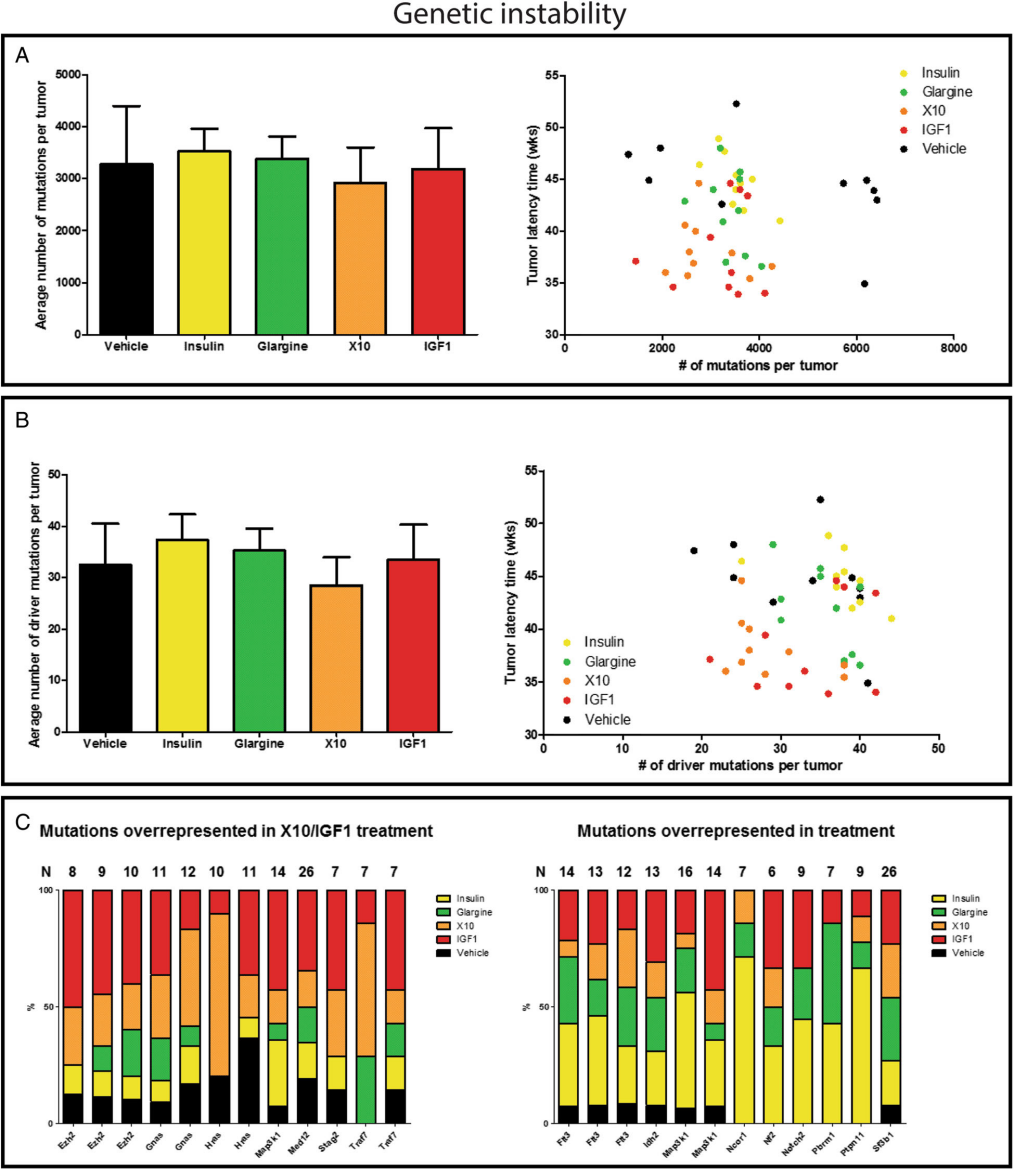
Genetic instability in mammary gland tumor tissue of chronically insulin analogue-exposed mice. **a** The bar plot shows the average number of mutations per MG tumor for all chronic treatments; the dot plot indicates that there is no correlation between the number of mutations of a specific tumor and tumor latency time. **b** The same as in **a**, but here we focus on clinically relevant human tumor driver mutations. **c** Some specific tumor driver mutations are featured in these bar plots. The first bar plot represents the mutations that are enriched in the X10/IGF1 treatment groups; in the second bar plot, the mutations are highlighted that are under-represented in the vehicle treatment group. *N* shows the number of tumors in which this specific mutation was present

Since IGF1 and X10 promote cell proliferation, this could lead to a manifestation of mutations and consequently modulation of tumor development and progression. To evaluate if chronic IGF1 or X10 treatment affects the number of mutations, a mutational analysis was performed on all tumors based on the 40 million 100 base pair reads for each tumor. The number of different mutations was ~3000 mutations per tumor and no significant difference between treatment groups could be detected. Furthermore, there was no correlation between the number of mutations per tumor and the tumor latency time (best fit correlation values, slope = 0.00001207, *r* ^2^ = 0.0000099, *P* value = 0.9827) (Fig. 6a).

Next, we focused on specific clinically relevant mutations that are part of the ~140 known human tumor drivers according to Vogelstein and colleagues [10]. In the 50 mouse tumors that we sequenced, 102 of these tumor drivers were mutated. On average, each tumor had about 35 of these tumor driver mutations and again it seems that the chronic treatment did not affect the overall number of tumor driver mutations per tumor (Fig. 6b). Also, for the tumor driver mutations, there was no correlation between the number of mutations and latency time of each tumor. Furthermore, no treatment-specific effects could be detected for the average number of point mutations, the average number of frame shifts, the average number of start CODON insertions, or the average number of stop CODON insertions (Additional file 2).

We next determined the mutations that are over-represented (>50%) in the X10/IGF1 treatments (Fig. 6c). All mutations are involved in cancer development and/or progression [10, 15]. Although no specific core pathway links these individual mutations, strikingly *Ezh2* and *Hras* were strongly over-represented in IGF1/X10-treated tumors. In a similar way we looked at specific mutations that have been over-represented in insulin analogue treatment altogether and highly under-represented in the vehicle condition. No general mechanism was identified that could explain the enrichment of these mutations in the treatment groups. However, a set of five mutations was not detected at all in the vehicle control, yet these were specifically affected in the insulin treatment group. Overall, these data suggest that the IGF1/X10 enhanced tumor formation is associated with several key candidate cancer drivers that could contribute, including *Ezh2* and *Hras* which are known drivers in human breast cancer [16, 17].

## Discussion

In this study, we used a next-generation sequencing-based transcriptome analysis to characterize mammary gland (MG) tumors from the p53^R270H/+^WAPCre mouse model that were chronically exposed to insulin-like molecules. We found indications that tumors of mice that received chronic treatment of X10 or IGF1 (compounds with a high affinity towards the IGF1R) show a transcriptomic profile that can be linked to a phenotype with an increased growth potential, enhanced migratory capabilities, and a higher Warburg potential. Moreover, the candidate cancer driver mutations in *Ezh2* and *Hras* were highly enriched in X10 and IGF1 tumors.

This is the first study in which a human relevant breast cancer mouse model has been used to study the tumorigenic effects of chronic insulin analogue treatment. Eventually, all mice from this model developed spontaneous MG tumors with a high human relevance. In this way it was possible to compare tumors induced by insulin analogue treatment with tumors induced by chronic insulin or vehicle treatment. This is in contrast to other studies using wild-type mice where only a few tumors with an origin less relevant to the human situation could be evaluated [18, 19].

Tumors often have an increased IRA:IRB ratio [20, 21] that can possibly influence the in vivo transformational effects of insulin analogues [22]. We found that IRA gene expression levels are upregulated (twofold) in pre-neoplastic MGs compared to expression levels in normal MGs, but IRA gene expression levels in tumors were similar to that of normal MG tissue. This might indicate that the IRA plays a stimulatory role in the transformation of normal to neoplastic MG tissue, but once the MG tumor is established the A isoform of the insulin receptor does not play a key role in proliferative signaling anymore. Surprisingly, IRB gene expression levels were strongly downregulated (over 10-fold) in MG tumors. This suggests that the IRA:IRB ratio is indeed increased in MG tumors, but this effect is mainly caused by downregulation of IRB expression levels rather than an upregulation of IRA.

We anticipated that chronic stimulation with insulin-like molecules would decrease the Warburg potency, as insulin deprivation in human fibroblasts led to an induction of anaerobic glycolysis [23]. Indeed, we saw that 90% of the insulin-induced tumors showed an increased oxidative phosphorylation response compared to 60% for the spontaneous vehicle-induced tumors. Interestingly, we found that compounds that induced an equi-glycemic response but with an increasing mitogenic potential at the doses used in this study (insulin, glargine, X10, and IGF1) [7] also showed an increasing percentage of tumors depending on anaerobic glycolysis (10%, 30%, 50%, and 60%, respectively). This might suggest that proliferative signaling is indirectly or directly coupled to the Warburg effect. Tumor samples have deliberately been taken 24 h after the last injection, since we were interested in the long-term rather than the short-term in vivo effects. During this time the exogenous compounds are fully degraded by enzymes and therefore no short-term signaling effect of the compounds can be observed [24, 25]. However, we cannot fully exclude the effect of direct insulin analogue treatment on tumor cell metabolism, since treatment of MCF7-IGF1R cells with the various insulin analogues also affected the glycolytic metabolic program.

Using the SVM model we could detect a sustained proliferative signaling in the chronic X10/IGF1-treated tumors which might suggest that chronic growth factor treatment can transform tumors in such a way that an autocrine growth factor signaling pathway is induced. A likely explanation would be a differential mutational pattern in cancer driver genes that underlie such a differential proliferative pathway. Chronic insulin, glargine, X10, or IGF1 treatment did not result in more mutations, and no correlation could be detected with the number of mutations or tumor latency time. Interestingly several X10/IGF1-enriched mutations were observed, including *Ezh2*, *Hras*, and *Traf7*, of which *Ezh2* and *Hras* are prominent modulators of human breast cancer. It is possible that these specific mutations contribute (in)directly to the X10/IGF1 phenotypes and enhanced tumor development and progression. Since we have only performed RNAseq we can, of course, not exclude other genomic mutations that were missed in our analysis and that could contribute to MG tumor development and explain the IGF1R-driven enhancement of tumor development/progression in our models.

## Conclusions

Altogether our data suggest that the observed decreased tumor latency time in the p53^R270H/+^WAPCre mouse model after chronic X10/IGF1 treatment is a result of an enhanced tumor biomass production rate. Furthermore, these treatments might facilitate tissue invasion and metastasis and deregulate the cellular metabolism in the tumor. All these factors contribute to an enhanced tumor development, thus decreasing the MG tumor latency time in this model. We did not find any evidence that chronic glargine treatment induced a more aggressive tumor phenotype or increased the biomass production rate, but a slight increased Warburg potential was observed compared to tumors induced by insulin treatment.

## Additional files

Additional file 1: Average number of MG tumors increased in chronic IGF1 treated mice. The average number of mammary gland tumors per treatment group. (TIF 2642 kb)
Additional file 2: Chronic insulin analogue treatment does not affect the mutational profile of the tumors. A) The average number of point mutations per treatment group. B) The average number of frame shifts per treatment. C) The average number of start CODON insertions per treatment. D) The average number of stop CODON insertions per treatment. (TIF 12348 kb)

## Abbreviations

EMT: Epithelial to mesenchymal transition
FDR: False discovery rate
IGF1: Insulin-like growth factor 1
IGF1R: Insulin-like growth factor 1 receptor
IPA: Ingenuity Pathway Analysis
IR: Insulin receptor
IRA: A isoform of IR
IRB: B isoform of IR
MET: Mesenchymal to epithelial transition
MeV: Multi-experiment viewer
MG: Mammary gland
MILP: Mixed integer linear programming
NGS: Next-generation sequencing
SMA: Smooth muscle actin
SNP: Single nucleotide polymorphism
SVM: Support vector machine
