# Supplementary figures and images for: Insulin-like growth factor 1 receptor activation promotes mammary gland tumor development by increasing glycolysis and promoting biomass production
Source: Breast Cancer Res. 2017 Feb 7;19:14. doi: 10.1186/s13058-017-0802-0 (PMC5297135; doi:10.1186/s13058-017-0802-0)

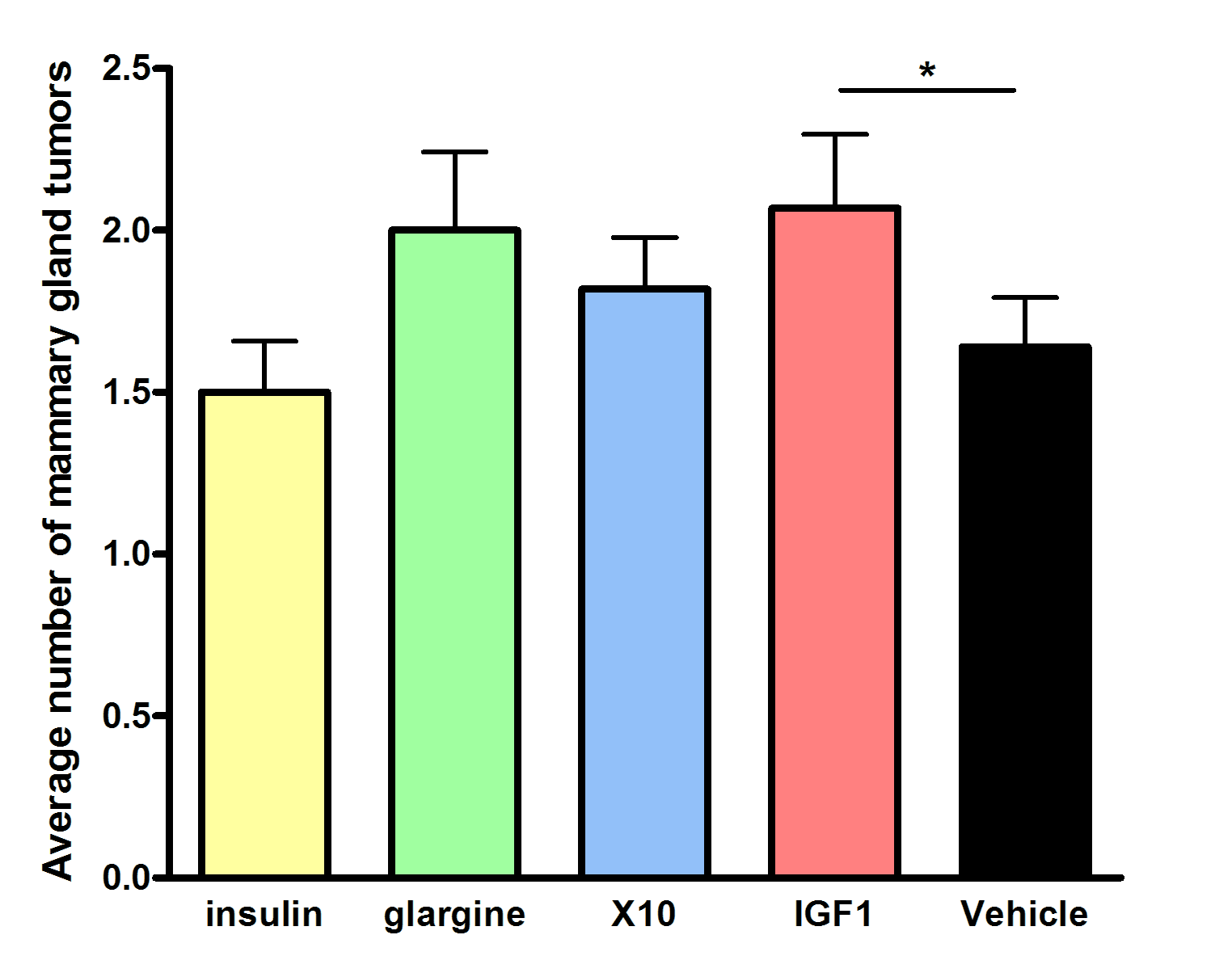

Supplement: Additional file 1: — Average number of MG tumors increased in chronic IGF1 treated mice. The average number of mammary gland tumors per treatment group. (TIF 2642 kb) [file 13058_2017_802_MOESM1_ESM.tif]

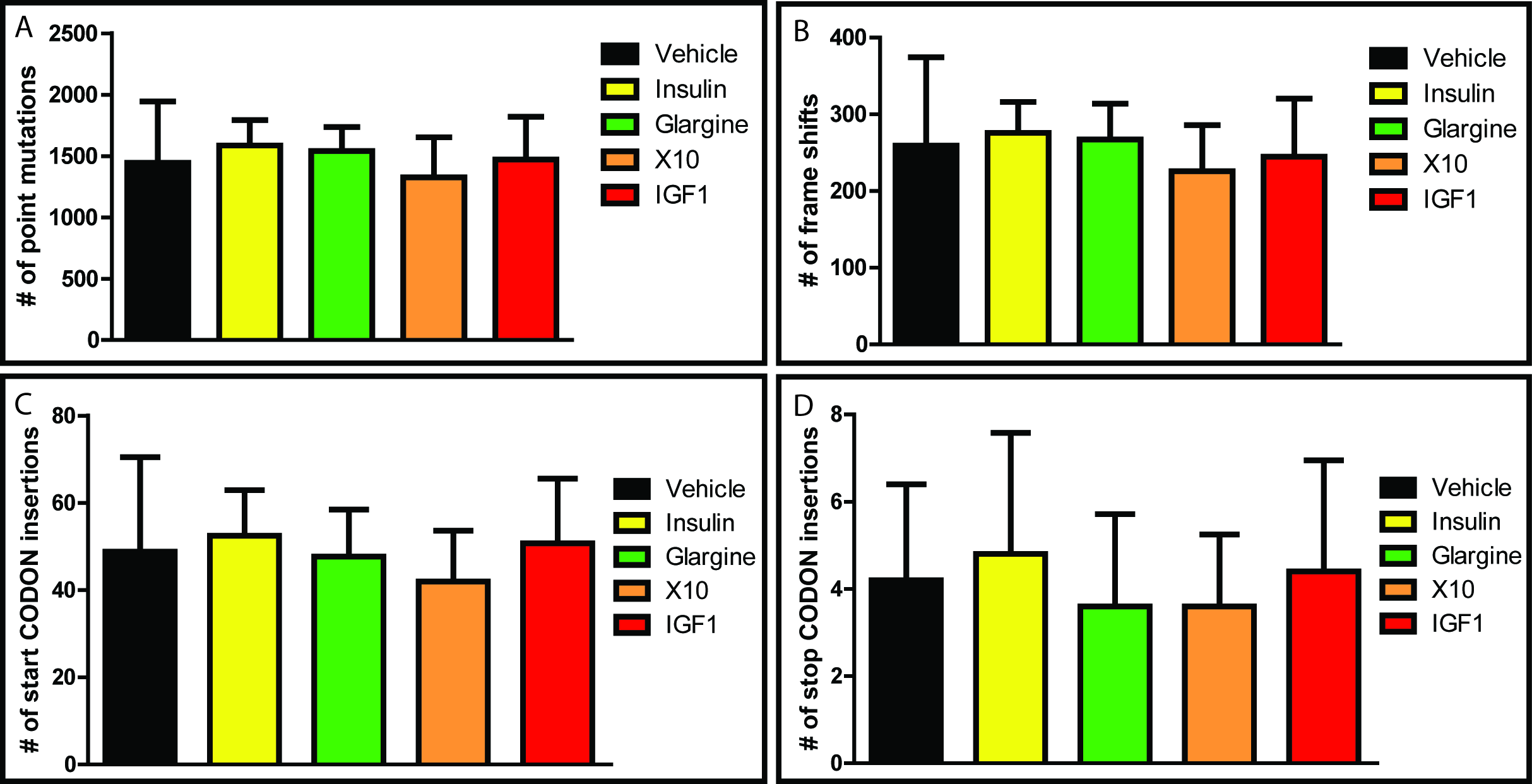

Supplement: Additional file 2: — Chronic insulin analogue treatment does not affect the mutational profile of the tumors. A) The average number of point mutations per treatment group. B) The average number of frame shifts per treatment. C) The average number of start CODON insertions per treatment. D) The average number of stop CODON insertions per treatment. (TIF 12348 kb) [file 13058_2017_802_MOESM2_ESM.tif]
